# Investigate the risk factors of stunting, wasting, and underweight among under-five Bangladeshi children and its prediction based on machine learning approach

**DOI:** 10.1371/journal.pone.0253172

**Published:** 2021-06-17

**Authors:** S. M. Jubaidur Rahman, N. A. M. Faisal Ahmed, Md. Menhazul Abedin, Benojir Ahammed, Mohammad Ali, Md. Jahanur Rahman, Md. Maniruzzaman

**Affiliations:** 1 Statistics Discipline, Khulna University, Khulna, Bangladesh; 2 Department of Statistics, University of Rajshahi, Rajshahi, Bangladesh; University of Western Australia, AUSTRALIA

## Abstract

**Aims:**

Malnutrition is a major health issue among Bangladeshi under-five (U5) children. Children are malnourished if the calories and proteins they take through their diet are not sufficient for their growth and maintenance. The goal of the research was to use machine learning (ML) algorithms to detect the risk factors of malnutrition (stunted, wasted, and underweight) as well as their prediction.

**Methods:**

This work utilized malnutrition data that was derived from Bangladesh Demographic and Health Survey which was conducted in 2014. The selected dataset consisted of 7079 children with 13 factors. The potential risks of malnutrition have been identified by logistic regression (LR). Moreover, 3 ML classifiers (support vector machine (SVM), random forest (RF), and LR) have been implemented for predicting malnutrition and the performance of these ML algorithms were assessed on the basis of accuracy.

**Results:**

The average prevalence of stunted, wasted, and underweight was 35.4%, 15.4%, and 32.8%, respectively. It was noted that LR identified five risk factors for stunting and underweight, as well as four factors for wasting. Results illustrated that RF can be accurately classified as stunted, wasted, and underweight children and obtained the highest accuracy of 88.3% for stunted, 87.7% for wasted, and 85.7% for underweight.

**Conclusion:**

This research focused on the identification and prediction of major risk factors for stunting, wasting, and underweight using ML algorithms which will aid policymakers in reducing malnutrition among Bangladesh’s U5 children.

## 1 Introduction

Malnutrition is one of the most serious health and welfare issues in any developing country, including Bangladesh. Malnutrition is referred to a lack of, excess, or imbalance in an individual’s energy and/or nutrient intake [1]. Malnutrition is a general term that refers to two distinct conditions as under-nutrition and overweight. Undernutrition includes stunting, wasting, and being underweight, whereas overweight/obesity is associated with a number of non-communicable diseases (diabetes, cancer, stroke, and heart disease) [1–3]. According to the WHO, approximately 1.9 billion adults worldwide were overweight, while 462 million were underweight. It was also noted that there were 47 million wasted U5 children, 14.3 million severely wasted, and 144 million stunted children, with 38.3 million overweight/obese children. Globally, 2.6 million children die per year due to malnutrition and 45% of U5 deaths were due to under-nutrition [4, 5].

In the case of Bangladesh, malnutrition affects more than half the population [6]. A total of 450,000 children suffered from severe acute malnutrition, while nearly 2 million suffered from mild acute malnutrition [7]. Children’s nutritional status has gradually improved over the last decades [8]. The prevalence of U5 stunted children was 51% in 2004, 43% in 2007, and 41% in 2011, while the prevalence of underweight children was 43% in 2004, 41% in 2007, and 36% in 2011. Similarly, 15% of U5 children were wasted in 2004, 17% in 2017, and 16% in 2011 [8]. These figures declined to 36% stunted, 33% underweight, and 14% overweight in 2014 [9]. The height-for-age (stunted), weight-for-height (wasted), and weight-for-age (underweight) were widely used to decide whether or not a child was malnourished [9, 10].

Child malnutrition is a hot topic in the field of public health as well as epidemiology globally. There were lots of studies about U5 malnutrition around the world. In previous studies, they focused only on the identification of the risk factors of malnutrition using classical model like logistic regression [1–3, 11–21]. Therefore, it is necessary to propose a predictive model on the identified the significant risk factors for predicting malnutrition. Nowadays, machine learning (ML) has great attractions for predicting different types of medical/biomedical data. Recently, the applications of ML in the field of public health have increased day by day. Some works on ML were used for prediction of different fields as malnutrition [22–24], anemia [25–27], diabetes [28], low birth weight [29–32], child mortality [33–35], and so on. There was also some work on ML for prediction of underweight [22–24, 36, 37], stunted and wasted [23, 24]. In the previous studies, they did not tune the hyper-parameters of ML algorithms. As a result, their ML algorithm performance did not give any satisfactory accuracy. The hypothesis of this work is to propose a combination of logistic regression (LR) based risk factor identification method along with ML classifiers to more accurately classify malnutrition and yield the highest accuracy. To support this claim, we have used support vector machine (SVM), random forest (RF), and LR for predicting malnutrition and compared their performances were assessed by accuracy and area under the curve (AUC).

## 2 Materials and methods

### 2.1 Dataset and study design

This work utilized malnutrition data that has been derived from BDHS, 2014 which was conducted in 2014 and freely available online. It was the 7^th^ nationwide DHS, covering the entire population. The list of enumeration areas (EA) of the 2011 census population was provided by the bureau of statistics (BBS). The samples of households of BDHS, 2014 were collected using two-stage stratified sampling. In the 1st stage, 600 EAs were chosen at random, proportional to their number, and only 30 households were chosen using systematic sampling. Approximately 18, 000 ever-married women (age: 15–49 years) were selected for an interview and 17,863 (99%) women were successfully interviewed [8]. We have used a kid’s recode file from BHDS, 2014, comprised of 7886 respondents. A total of 7079 respondents were selected after eliminating 807 missing values for the final analysis.

### 2.2 Ethical approval

This study was based on an analysis of existing public domain survey datasets that are freely available online with all identifier information removed. The survey was approved by the Ethics Committee in Bangladesh. The authors were granted permission to use the data for independent research purposes.

### 2.3 Response variable and explanatory variables

In this work, we have considered three types of response variables as stunted, underweight, and wasted, which were measured based on height-for-age Z-score (HAZ), weight-for-height Z-score (WHZ), and weight-for-age Z-score (WAZ). Using WHO AnthroPlus (version 3.2.2, 2011), the Z-scores were determined on the basis of age, weight, and height [38]. Children were considered as stunted if HAZ≤-2 standard deviation (SD). Similarly, wasted and underweight were defined as WAZ≤-2 SD, and WHZ≤-2 SD [39]. Various socio-economic and demographic factors were chosen as explanatory variables based on the literature [11–21]. The brief descriptions of the selected explanatory variables along with their categories were discussed in Table 1.

**Table 1:** Prevalence of stunting, wasting and underweight.

Factors	Categories	Total, n (%)	Stunted, n (%)	p-value^1^	Wasted, n (%)	p-value^1^	Underweight, n (%)	p-value^1^
**Total**			2506 (35.4)		1090 (15.4)		2322 (32.8)	
Region	Barisal	830(11.7)	261(31.4)	<0.001	157(18.9)	<0.001	276(33.3)	<0.001
	Chittagong	1347(19)	565(41.9)		214(15.9)		446(33.1)	
	Dhaka	1236(17.5)	360(29.1)		161(13.0)		320(25.9)	
	Khulna	787(11.2)	180(22.9)		113(14.4)		179 (22.7)	
	Rajshahi	883(12.5)	255(28.9)		158(17.9)		272(30.8)	
	Rangpur	874(12.3)	270(30.9)		144(16.5)		320(36.6)	
	Sylhet	1122(15.8)	615(54.8)		143(12.7)		509(45.4)	
Type of place	Urban	2229(31.5)	639(28.7)	<0.001	289(13.0)	<0.001	581(26.1)	<0.001
	Rural	4850(68.5)	1867(38.5)		801(16.5)		1741(35.9)	
Sex	Male	3639(51.4)	1261(34.7)	0.439	596(16.4)	0.019	1168(32.1)	0.431
	Female	3440(48.6)	1245(36.2)		494(14.4)		1154(33.5)	
Child’s age (years)	≤ 1	2881 (40.7)	375(13.0)	<0.001	517 (18.0)	<0.001	412(14.3)	<0.001
	>1	4198 (59.3)	2131(50.8)		573 (13.6)		1910(45.5)	
Mother’s education	No education	1093(15.4)	627(57.4)	<0.001	175(16.0)	0.009	525(48.0)	<0.001
	Primary	1963(27.7)	867(44.2)		341(17.4)		827(42.1)	
	Secondary	3276(46.3)	887(27.1)		478(14.6)		854(26.1)	
	Higher	747(10.6)	125(16.7)		96(12.9)		116(15.5)	
Father’s education	No education	1762(24.9)	965(54.8)	<0.001	290(16.5)	0.024	876(49.7)	<0.001
	Primary	2134(30.1)	842(39.5)		356(16.7)		772(36.2)	
	Secondary	2155(30.4)	546(25.3)		303(14.1)		509(23.6)	
	Higher	1028(14.5)	153(14.9)		141(13.7)		165(16.1)	
Mother’s age (year)	12–18	4272(60.3)	1689(39.5)	<0.001	702(16.4)	0.009	1572(36.8)	<0.001
	19–35	2798(39.5)	812(29.0)		386(13.8)		745(26.6)	
	35–49	9(0.1)	5(55.6)		2(22.2)		5(55.6)	
Mother’s working status	No	5315(75.1)	1787(33.6)	0.003	804(15.1)	0.273	1642(30.9)	<0.001
	Yes	1764(24.9)	719(40.8)		286(16.2)		680(38.5)	
Birth order	1^st^ birth	2743(38.7)	782(28.5)	<0.001	420(15.3)	0.795	762(27.8)	<0.001
	2^nd^ birth	2128(30.1)	749(35.2)		321(15.1)		636(29.9)	
	Others	2208(31.2)	975(44.2)		349(15.8)		924(41.8)	
Twin child	Single birth	6998(98.9)	2463(35.2)	0.113	1079(15.4)	0.983	2287(32.7)	0.291
	1^st^ of multiple	46(0.6)	28(60.9)		7(15.2)		23(50.0)	
	2^nd^ of multiple	35(0.5)	15(42.9)		4(11.4)		12(34.3)	
Drinking water	Safe source	6321(89.3)	2230(35.3)	0.772	956(15.1)	0.066	2083(33.0)	0.647
	Unsafe source	758(10.7)	276(36.4)		134(17.7)		239(31.5)	
Toilet types	Hygienic	6064(85.7)	2052(33.8)	<0.001	909(15.0)	0.020	1916(31.6)	0.001
	Unhygienic	1015(14.3)	454(44.7)		181(17.8)		406(40.0)	
Wealth index	Poor	2873(40.6)	1383(48.1)	<0.001	532(18.5)	<0.001	1324(46.1)	<0.001
	Middle	1399(19.8)	481(34.4)		212(15.2)		441(31.5)	
	Rich	2807(39.7)	642(22.9)		346(12.3)		557(19.8)	

^1^p-value is obtained from the Chi-Square test.

### 2.4 Statistical analysis

All categorical data was expressed as number (%). The chi-square analysis was implemented to assess the relationship between various selected explanatory variables and malnutrition (stunted, wasted, and underweight). If the explanatory variables were statistically significantly associated with malnutrition, these significant variables were fed to LR model. LR based model was implemented to determine the risk factors of malnutrition. The significant risk factors were selected on the basis of p-value (p<0.05). Then three well-known and popular ML algorithms which were available in literature as support vector machine (SVM) [40], logistic regression (LR) [41], and random forest (RF) [42] were implemented for predicting malnutrition status. STATA version 14 and R i386 4.0.0 were used for all statistical analyses.

### 2.5 Overview of machine learning system

**Fig 1:**
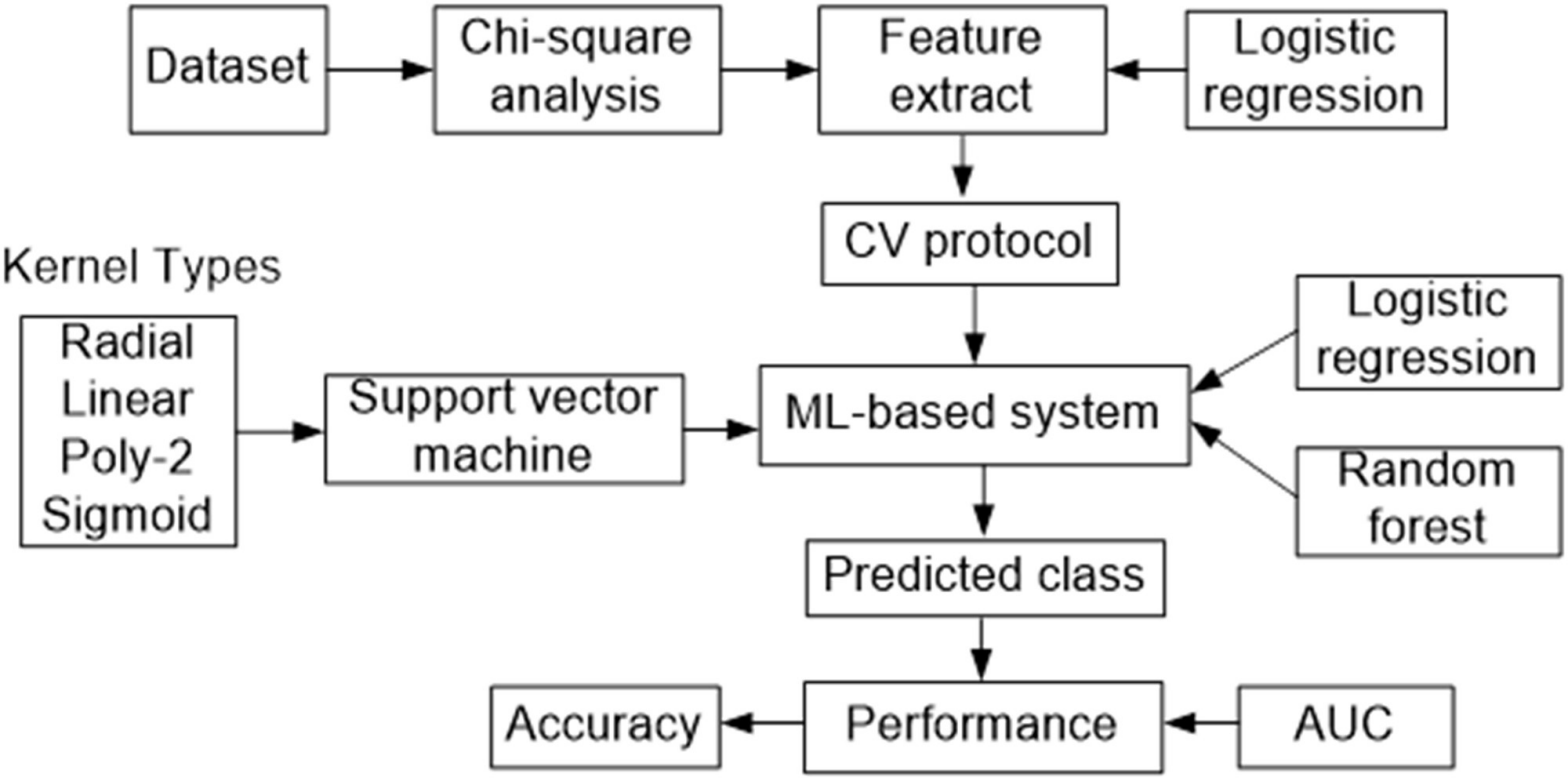
Overview of machine learning-based study.

The overview of ML-based study was depicted in Fig 1. The chi-Square analysis was adopted to determine the relationship between various explanatory variables and malnutrition. LR was implemented and selected risk factors of malnutrition using p-value (p<0.05). Then, we adopted 10-fold cross-validation as well as three ML algorithms as SVM, RF, and LR for predicting malnutrition. In this work, we used radial basis function (RBF), linear, polynomial (Poly-2), and sigmoid kernels of SVM. We optimized the best kernel for SVM on the basis of accuracy and AUC and compared its performance with RF and LR.

## 3 Results

### 3.1 Baseline and demographic characteristic of respondents

Table 1 shows the respondents’ baseline and demographic characteristics. The average prevalence of stunted, wasted, and underweight was 35.4%, 15.4%, and 32.8%, respectively, as shown in Table 1. The region was significantly associated with stunted, wasted, and underweight children. The largest number of stunted (54.8%) and underweight (45.4%) children were from the Sylhet region, while 15.9% wasted children from the Chittagong. Whereas, the lowest number of stunted (11.2%), wasted (10.4%), and underweight (22.7%) were from the Khulna region and wasted (12.7%) from Sylhet region. Most of the malnourished children came from rural areas. It was noticed that region, type of place, fathers and mother’s education, mother’s and child’s age, toilet types, and wealth index were significantly linked to stunted, wasted, and underweight. It was also noticed that mothers’ occupations and birth order were associated with stunted and underweight children, whereas a child’s sex was only statistically associated with wasted.

### 3.2 Risk factors extraction using logistic regression

Table 2 depicts the effect of various associated factors on stunted, wasted, and underweight using LR. According to the LR findings, five factors (region, father’s age, child’s age, toilet types, and wealth index) were statistically significant for stunted and underweight children, while four factors (region, child’s age and sex, and wealth index) were statistically significant for wasted children (see Table 2). These factors were considered risk factors for malnutrition because their p-value was less than 0.05.

**Table 2:** Risk factors extraction of stunted, wasted, and underweight using LR.

Factors	Categories	Stunted	Wasted	Underweight
		OR (95% CI)	OR (95% CI)	OR (95% CI)
Region	Barisal	0.58 (0.44–0.75) *	1.56 (1.21–2.02) *	0.77 (0.60–1.00) *
	Chittagong	0.91 (0.73–1.14)	1.40 (1.11–1.77) *	0.86 (0.69–1.07)
	Dhaka	0.61 (0.48–0.78) *	1.11 (0.86–1.42)	0.68 (0.53–0.86) *
	Khulna	0.45 (0.33–0.60) *	1.20 (0.91–1.58)	0.55 (0.42–0.74) *
	Rajshahi	0.48 (0.37–0.64) *	1.5 (1.16–1.93) *	0.67 (0.52–0.86) *
	Rangpur	0.51 (0.39–0.66) *	1.31 (1.01–1.69) *	0.79 (0.61–1.01)
	Sylhet^®^			
Type of place	Urban	1.03 (0.86–1.23)	0.88 (0.75–1.03)	1.04 (0.88–1.23)
	Rural^®^			
Sex of child	Male	0.96 (0.83–1.10)	1.17 (1.03–1.33) *	0.96 (0.84–1.10)
	Female^®^			
Age of child (years)	≤ 1	0.15 (0.13–0.16)	1.38(1.22–1.58)	0.20(0.18–0.23)*
	>1^®^			
Mother’s education	No education	1.23 (0.81–1.86)	1.14 (0.81–1.62)	1.21 (0.81–1.81)
	Primary	1.08 (0.73–1.60)	1.23 (0.99–1.68)	1.30 (0.89–1.88)
	Secondary	0.87 (0.61–1.26)	1.08 (0.82–1.42)	1.06 (0.75–1.50)
	Higher^®^			
Father’s education	No education	2.25 (1.55–3.24) *	0.91 (0.69–1.22)	1.71 (1.23–2.38) *
	Primary	1.98 (1.39–2.82) *	0.94 (0.72–1.22)	1.45 (1.05–1.99) *
	Secondary	1.55 (1.10–2.18) *	0.86 (0.68–1.10)	1.19 (0.88–1.61)
	Higher^®^			
Mother’s age	12–18	0.59 (0.09–3.74)	0.68 (0.14–3.34)	0.62 (0.10–3.74)
	19–35	0.51 (0.08–3.24)	0.61 (0.12–2.99)	0.54 (0.09–0.30)
	35–49			
Mother’s working status	No	0.94 (0.80–1.11)	0.93 (0.80–1.08)	0.94 (0.81–1.10)
	Yes^®^			
Birth order	1^st^ birth	1.01 (0.84–1.22)	1.04 (0.88–1.24)	0.93 (0.78–1.11)
	2^nd^ birth	1.17 (0.97–1.41)	1.01 (0.84–1.19)	0.92 (0.77–1.10)
	Others^®^			
Twin child	Single birth	0.74 (0.29–1.86)	0.98 (0.37–2.56)	1.08 (0.40–2.91)
	1^st^ of multiple	1.47 (0.46–4.75)	1.06 (0.30–3.72)	1.91 (0.56–6.49)
	2^nd^ of multiple^®^			
Source of drinking water	Safe source	0.93 (0.70–1.24)	0.90 (0.68–1.18)	1.10 (0.76–1.32)
	Unsafe source^®^			
Types of toilet	Hygienic	0.77 (0.70–1.24) *	0.97 (0.76–1.24)	0.79 (0.63–1.00) *
	Unhygienic^®^			
Wealth index	Poor	1.77 (1.43–2.20) *	1.38 (1.14–1.68) *	2.02 (1.65–2.49) *
	Middle	1.40 (1.12–1.76) *	1.13 (0.93–1.39)	1.49 (1.20–1.85) *
	Rich^®^			

^®^: Reference category;

* Indicates significant at 5% level of significance.

### 3.3 Kernel selection of SVM

There are various kernels in SVM. As a result, the kernel of SVM must be optimized. In this work, we implemented SVM with 4 kernels: linear, RBF, Poly-2, and sigmoid. We tuned the hyper-parameters of these kernels using grid search methods. We optimized the kernel based on accuracy and chose the kernel with the highest accuracy. It was observed that RBF kernel provided the highest accuracy of 88.1% for stunted, 86.0% for wasted, and 85.6% for underweight compared to other kernels (see Table 3). That is why, RBF kernel was chosen for the SVM to predict stunted, wasted, and underweight children.

**Table 3:** SVM Kernel selection based on accuracy (%).

Kernel types	Stunted	Wasted	Underweight
Linear	85.5	84.6	83.2
**RBF**	**88.1**	**86.0**	**85.6**
Poly-2	86.9	84.2	81.1
Sigmoid	85.6	83.5	82.6

The bolded value represents the proposed method results.

### 3.4 Comparison of the efficiency of ML algorithms

Accuracy and AUC were used to evaluate the efficiency of ML algorithms. Since the BDHS dataset was categorical, it was a very tedious task to choose a predictive model that could be accurately classified with the highest accuracy and AUC. The comparison of the efficiency of ML algorithms is depicted in Table 4. It was noted that the highest accuracy of 88.3% for stunted, 87.7% for wasted, and 85.7% was achieved by SVM with RBF kernel, while LR classifier provided the accuracy of 87.7% for stunted, 83.6% for wasted, and 84.5% for underweight. As a result, it was concluded that the RF classifier outperformed the LR and SVM for predicting stunted, wasted, and underweight children.

**Table 4:** Comparison of accuracy (%) of ML algorithms.

Classifier types	Stunted	Wasted	Underweight
LR	87.7	83.6	84.9
SVM	88.1	86.0	85.6
**RF**	**88.3**	**87.7**	**85.7**

The bolded value represents the proposed method results.

The AUC of the ML algorithms was presented in Table 5. It was clearly noted that RF classifier achieves the highest AUC of 0.714 for stunted, 0.523 for wasted, and 0.664 for underweight compared to LR and SVM.

**Table 5:** Comparison of AUC of ML algorithms.

Classifier types	Stunted	Wasted	Underweight
LR	0.602	0.518	0.577
SVM	0.610	0.519	0.581
**RF**	**0.714**	**0.523**	**0.664**

The bolded value represents the proposed method results.

## 4 Discussion

The goal of this research was to identify risk factors for malnutrition and predict it using ML algorithms. Previously, only two studies on ML-based prediction of malnutrition status were conducted in Bangladesh, but they had lower accuracy [23, 24]. In this work, LR model was implemented to determine the risk factors of malnutrition (stunted, wasted, and underweight) on the basis of p-value (p<0.05). According to LR findings, five factors (region, child’s age, father’s education, toilet types, and wealth index) were statistically significant risk factors for stunted and underweight children, while four factors (region, child’s age and sex, and wealth index) were also significant risk factors for wasted children. Our findings showed that the children who came from Barisal, Chittagong, Dhaka, and Khulna were found to have a higher risk of wasted compared to the children who came from Sylhet region. The previous research also found that Bangladesh region had higher risk factors of wasted [2, 10, 14]. The sex of child was also a significant risk predictors of wasted, with male children having 1.17 times higher risk of wasted compared to female children. This finings was also coincided with previous studies [2, 10, 43].

Male children historically provided more parental attention. This has recently changed. The government of Bangladesh has adopted some polices, including stipends and free education to improve female education. Our findings also revealed that the children whose father’s had no education, only primary and secondary education, were at a higher risk of being wasted and underweight than children whose father had a higher education [44]. This study also illustrated that the wealth index had a significant impact on stunted, wasted, and underweight children. The poor family’s children had a higher chance of being stunting, wasting and underweight children compared to the rich family’s children, which was also consisted with previous studies [2, 45, 46]. The significant factors which were obtained from LR were fed into three ML algorithms (LR, SVM, and RF) to predict stunted, wasted, and underweight children. We need to optimize the kernel of SVM on the basis of accuracy from four kernels: linear, RBF, Poly-2, and sigmoid. Our findings showed that SVM with RBF kernel outperformed other methods for predicting stunted, wasted, and underweight children. Then, we used 10-fold CV as well as SVM with RBF kernel, RF, and LR implemented for predicting stunted, wasted, and underweight children. Finally, it may be concluded that the highest accuracy and AUC for stunted, wasted, and underweight were obtained by RF classifier.

### 4.1 Key difference between our research and previous research in literature

Many researches have been conducted on U5 malnutrition around the world. Among these few studies, there were two studies performed on stunted, wasted, and underweight [23, 24] and others on underweight [22–24, 36, 37]. In 2014, a cross-sectional study was conducted in 2014 in India to predict underweight children using ML algorithms. They implemented three types of classifiers: multilayer perceptron (MLP), RF, and ID3 and77.2% accuracy was provided by RF [22]. Kuttiyapillai & Ramachandrn [36] implemented SVM, artificial neural network (ANN), and k-nearest neighborhood (KNN) for predicting underweight. The highest accuracy of 94.7% was obtained by ANN. Mani & Kasireddy [37] also conducted a study on 145263 respondents in 2014 in America. They also implemented LR, RF, and linear discriminant analysis (LDA), and RF for predicting underweight. Shahriar et al. [23] applied SVM, ANN, and decision tree (DT), naïve Bayes (NB), and RF for predicting stunted, wasted, and underweight. They showed that ANN provided the highest accuracy of 67.3% for stunted, 86.0% for wasted and 70.0% for underweight. Talukder and Ahammed [24] also applied RF, SVM, LR, LDA, and k-NN for predicting underweight and they presented that RF obtained higher accuracy of 68.5%. For this work, it is observed that RF classifier achieves the largest accuracy of 88.3% for stunted, 87.7% for wasted and 85.7% for underweight, which are shown in Table 6. So, it can be concluded that RF is better than SVM and LR.

**Table 6:** Key difference between our research and previous research published in literature.

Authors	Year	Data size	Country	FS	Classifier types	Accuracy (%)		
						Stunted	Wasted	Underweight
Thangamani & Sudha [22]	2014	254	India	NA	ID3, **RF**, MLP	NA	NA	77.2
Kuttiyapillai & Ramachandrn [36]	2014	150	India	NA	**ANN**, SVM, KNN	NA	NA	94.7
Mani & Kasireddy [37]	2018	145263	America	NA	MLR, LDA, **RF**	NA	NA	86.3
Shahriar et al. [23]	2019	6995	Bangladesh	NA	**ANN**, SVM, RF, NB, DT	67.3	86.0	70.0
Talukder & Ahammed [24]	2020	6868	Bangladesh	NA	LDA, k-NN,SVM, **RF**, LR	NA	NA	68.5
Our Study	2021	7079	Bangladesh	LR	SVM, **RF**, LR	**88.3**	**87.7**	**85.7**

### 4.2 Strengths, limitations, and future recommendations

The main strength of this work is to extract high-risk factors of stunted, wasted, and underweight using a logistic regression model and make a decision based on the p-value. We used three ML algorithms (SVM, LR, and RF) to predict stunted, wasted, and underweight children. Among them, RF-based classifier outperformed comparison to previous studies published in the literature. This work has some limitations. This work was only conducted on BHDS, 2014 cross-sectional data and no any post hoc analysis like Bonferroni correction was performed. In the future, we would like to consider pooled data as well as more factors to get precise results. We will also use principal component analysis, Fisher discriminant analysis, and mutual information for feature extraction of stunted, wasted, and underweight. We also attempt to use more ML algorithms in conjunction with deep learning classifiers and compare their results to this current work.

## 5 Conclusion

Malnutrition is one of the most serious health and welfare issues in Bangladesh. The prevalence and risk factors of stunted, wasted, and underweight were investigated in this work and their status predicted using ML algorithms. LR results illustrated that five factors (region, child’s age, father’s education, and toilet types, and wealth index) were statistically significant for stunted and underweight, while four factors (region, child’s age and sex, and wealth index) for wasted. Results also indicated that RF classifier obtained the highest accuracy of 88.3% for stunted, 87.7% for wasted and 85.73% for underweight. This work suggests that LR-RF based combination may be accurately classified and predict stunted, wasted, and underweight and yield higher accuracy.
